# In Vitro Induction of Tendon-Specific Markers in Tendon Cells, Adipose- and Bone Marrow-Derived Stem Cells is Dependent on TGFβ3, BMP-12 and Ascorbic Acid Stimulation

**DOI:** 10.3390/ijms20010149

**Published:** 2019-01-03

**Authors:** Carlotta Perucca Orfei, Marco Viganò, John R. Pearson, Alessandra Colombini, Paola De Luca, Enrico Ragni, Leonor Santos-Ruiz, Laura de Girolamo

**Affiliations:** 1 IRCCS Istituto Ortopedico Galeazzi, Orthopaedic Biotechnology Lab, 20161 Milan, Italy; carlotta.perucca@gmail.com (C.P.O.); alessandra.colombini@grupposandonato.it (A.C.); deluca.paola@grupposandonato.it (P.D.L.); enrico.ragni@grupposandonato.it (E.R.); laura.degirolamo@grupposandonato.it (L.d.G.); 2 Andalusian Centre for Nanomedicine and Biotechnology, BIONAND, 29590 Málaga, Spain; jrpearson@bionand.es (J.R.P.); lsantos@uma.es (L.S.-R.); 3 Network Centre for Biomedical Research – Biotechnology, Biomaterials and Nanomedicine, CIBER-BBN, 50018 Zaragoza, Spain; 4 Department of Cell Biology, Genetics and Physiology, Instituto de Investigación University of Málaga, 29016 Malaga, Spain

**Keywords:** tenogenic differentiation, adipose-derived MSCs, Bone marrow-derived MSCs, tendon cells, BMP-12, ascorbic acid, TGFbeta, FGF

## Abstract

Mesenchymal Stem Cells (MSCs) and tissue-specific progenitors have been proposed as useful tools for regenerative medicine approaches in bone, cartilage and tendon-related pathologies. The differentiation of cells towards the desired, target tissue-specific lineage has demonstrated advantages in the application of cell therapies and tissue engineering. Unlike osteogenic and chondrogenic differentiation, there is no consensus on the best tenogenic induction protocol. Many growth factors have been proposed for this purpose, including BMP-12, b-FGF, TGF-β3, CTGF, IGF-1 and ascorbic acid (AA). In this study, different combinations of these growth factors have been tested in the context of a two-step differentiation protocol, in order to define their contribution to the induction and maintenance of tendon marker expression in adipose tissue and bone marrow derived MSCs and tendon cells (TCs), respectively. Our results demonstrate that TGF-β3 is the main inducer of scleraxis, an early expressed tendon marker, while at the same time inhibiting tendon markers normally expressed later, such as decorin. In contrast, we find that decorin is induced by BMP-12, b-FGF and AA. Our results provide new insights into the effect of different factors on the tenogenic induction of MSCs and TCs, highlighting the importance of differential timing in TGF-β3 stimulation.

## 1. Introduction

Tendon disorders are a complex class of pathologies affecting a significant percentage of the worldwide population [1,2]. Current conservative treatments and pharmacological therapy with non-steroidal anti-inflammatory drugs are efficient in pain relief but fail to restore tissue homeostasis, so that in a high proportion of cases pathology progression eventually leads to surgical intervention, long rehabilitation periods and frequent re-occurrence of pain and disabilities [3].

Over recent decades the search for alternative treatments has increased, with Mesenchymal Stem Cells (MSCs) emerging as a valuable tool to improve the clinical outcome of tendon-related pathologies. Stem cell-based therapies are of particular interest due to their ability to both differentiate towards different cell lineages and promote a regenerative microenvironment through the secretion of bioactive molecules [4,5,6,7]. MSCs have varying differentiation abilities depending on their tissue of origin [8,9], thus identifying the most suitable source of MSCs for this application is a crucial first step towards achieving an efficient and stable induction of the tenogenic lineage. Tendon stem/progenitor cells (TSPCs), residing in a very specific tendon niche in vivo, may be more prone to tenogenesis than other types of MSCs [10], potentially representing the best MSC source for cell-based tendon therapies. Nevertheless, the low number of TSPCs present within the whole tendon cell (TC) population and their phenotypic drift following in vitro expansion account for the search for alternative MSC sources. Compared to TSPCs, bone marrow- and adipose- derived stem cells (BMSCs and ASCs, respectively) are available in larger amounts and represent the most widely used MSC types [11].

Preclinical studies have shown that BMSCs are able to improve histological and biomechanical properties in different animal models (rat, rabbit, horse and sheep) of tendon injury induced by surgical manipulation or collagenase administration [12,13], as well as in a naturally-occurring digital flexor tendinopathy in horses [14]. However, the direct use of BMSCs at the target site in vivo can lead to the formation of ectopic bone or cartilage, and the biomechanical strength of repaired tendons is often unsatisfactory [12,15,16]. Similarly, ASCs have shown promising results in the enhancement of tendon repair and regeneration in different animal models [17,18,19,20], but effective strategies to prevent their commitment towards adipogenic differentiation remain to be fully investigated [21].

A possible way of improving cell-based tendon therapies is to perform an initial priming step using growth factors to direct MSC populations toward the tenogenic lineage. However, a definitive in vitro protocol able to pre-condition MSCs in this context has yet to be defined. Different literature reports have described tenogenic induction protocols using Bone Morphogenetic Protein-12 (BMP-12) alone or combined with Connective Tissue Growth Factor (CTGF) and ascorbic acid (AA) [4,22,23,24,25,26]. However, these protocols often only resulted in an unsatisfactory increase of tenogenic marker expression. The Transforming Growth Factor-β (TGF-β) superfamily, especially TGF-β3, is also involved in tenogenic differentiation and is considered a tenogenic inducer both alone [27] and combined with other growth factors such as basic-Fibroblast Growth Factor (b-FGF) [28] and Insulin-like Growth Factor-1 (IGF-1) [29]. 

The high variability of protocols described in the literature is also related to the timing of tenogenic induction. While some studies adopted a constant biochemical stimulation for up to 14 days [27,30,31], others only apply a short induction with tenogenic factors exerting visible effects at later stages [4,32,33]. The results obtained in these studies convinced us to use a short induction phase.

In order to deepen our understanding of the tenogenic ability of BMP-12, TGF-β3, IGF-1, CTGF, b-FGF and AA, in this work, different combinations of these growth factors were applied in the same experimental setting. Moreover, the tenogenic potential of MSCs from different origins was investigated in order to identify the most suitable cell source for future regenerative medicine-based treatments of tendon disorders. 

## 2. Results

### 2.1. Scleraxis and Decorin Expression-Based Screen for Different Combinations of Candidate Tenogenic Media

**Figure 1:**
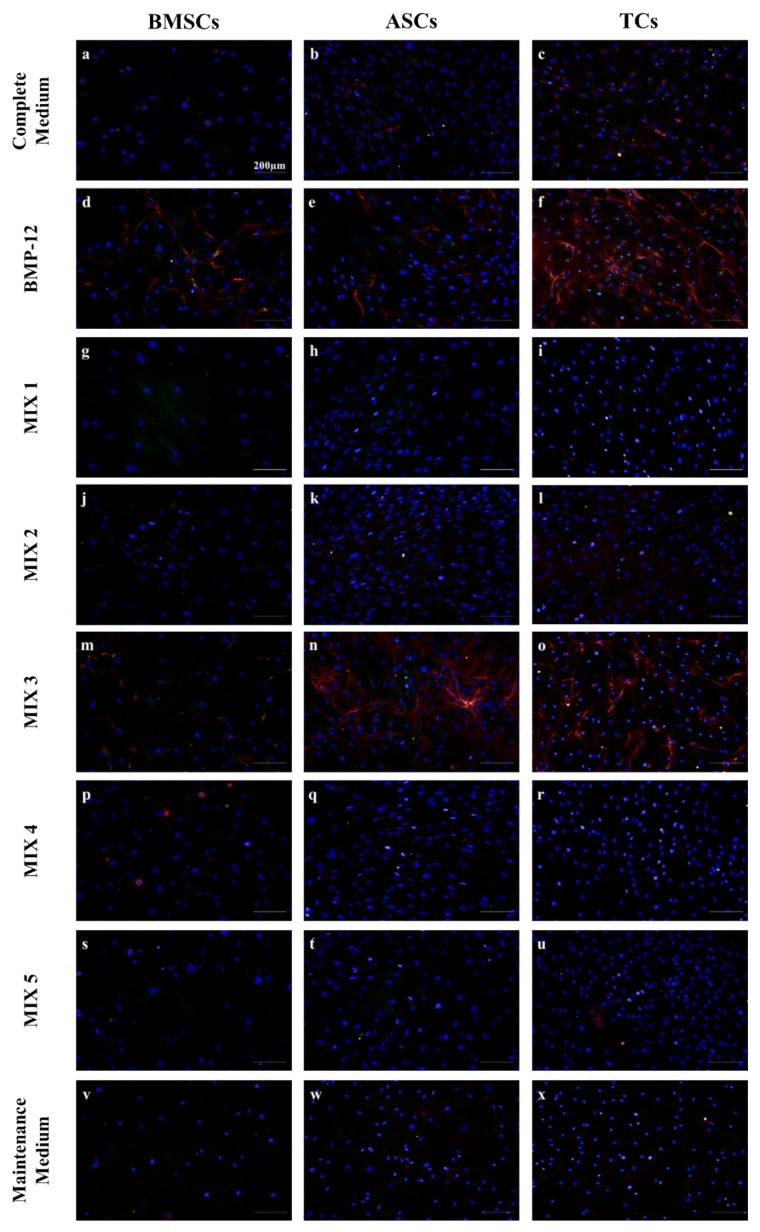
SCX and DCN immunofluorescence assays of BMSCs, ASCs and TCs cultured in different tenogenic media. Representative micrographs of BMSCs (a-d-g-j-m-p-s-v), ASCs (b-e-h-k-n-q-t-w) and TCs (c-f-i-l-o-r-u-x) after three days of culture in different tenogenic media. Cells were stained for DCN (red), SCX (green) and DNA (Blue). Scale bars = 200 µm.

After 3 days of tenogenic induction, the presence of decorin (DCN) and scleraxis (SCX) was assessed in cells cultured with different growth factor combinations (Table 1). As expected, BMP-12 was able to induce the expression of DCN in all cell types analyzed, which was visible in the form of red filaments when observed by fluorescence microscopy (Figure 1d–f). Similar accumulations of red DCN filaments were observed in all cell types treated with BMP-12 supplemented/TGF-β3 free medium (MIX 3) (Figure 1m–o). The effect of MIX 3 was especially strong on ASCs, which showed an evident increase in DCN filaments after three days of culture (Figure 1n), while weaker increases in DCN signal were seen in BMSCs and TCs (Figure 1m,o). In contrast, TGF-β3 enriched media (MIX 1, 2, 4 and 5) had little or no effect on DCN filament formation in all cell types after 3 days of culture (Figure 1). Using advanced image analysis techniques (see Materials and Methods and Figure S1 for details) we were able to automatically quantify decorin filament formation as part of our high throughput imaging screen.

The same samples were analyzed in terms of SCX distribution. Overall, variable SCX expression was observed between experiments, with weak staining only slightly above background typically observed (Figure 1). The presence of SCX was identified in the nuclear compartment as fluorescent dots, confirming its supposed subcellular localization (Figure S2) [34]. The frequency of nuclear SCX dot formation was analyzed using automated image analysis software (Figure S3). The frequency, as well as the fluorescence intensity, of SCX nuclear spots was unaffected by the different treatments with respect to the controls. However, for both ASCs and BMSCs, the MIX 2 treatment (BMP-12 free medium) resulted in their lowest nuclear SCX frequency. Overall, cells cultured in all TGF-β3 supplemented media (MIX 1, 2, 4 and 5) behaved in a consistent way, showing little or no DCN signal at day 3. This observation suggests that the presence of TGF-β3 actively inhibits the production of DCN. Moreover, the lack of BMP-12 (MIX 2) resulted in slightly lower SCX signal in MSCs, leading to the exclusion of this medium from further analyses. IGF-1 free medium (MIX 4) did not show any improvement in the expression of SCX and DCN, and therefore was not considered in subsequent gene expression analysis experiments. 

### 2.2. Role of b-FGF and Ascorbic Acid in the Maintenance of DCN Expression

**Figure 2:**
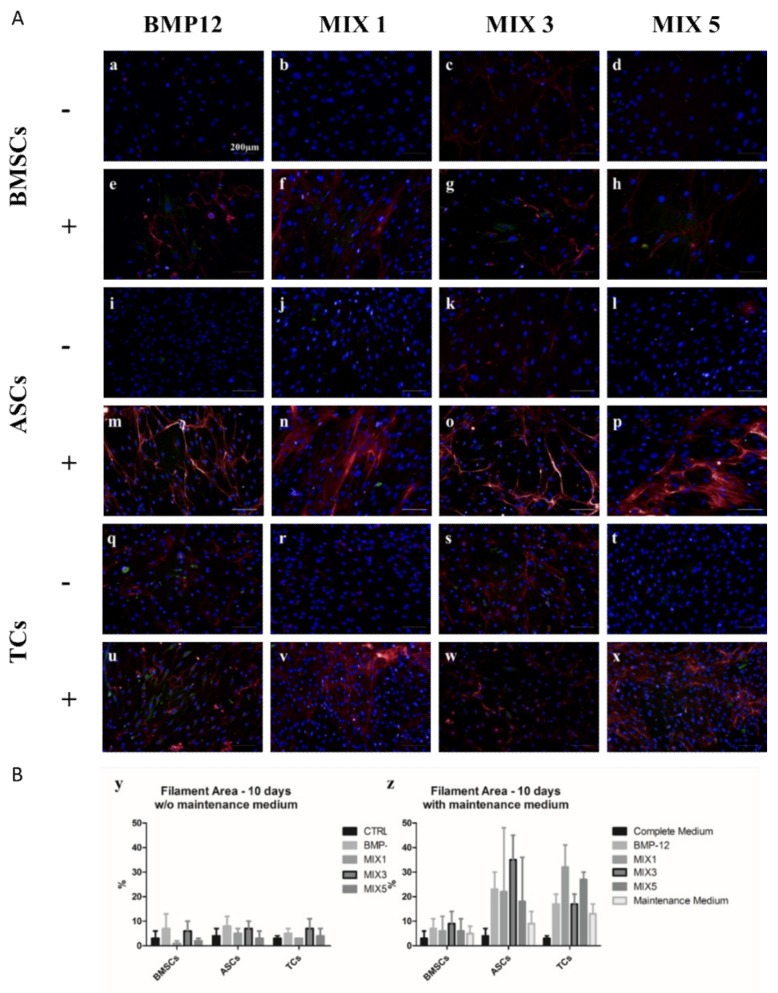
DCN expression in the presence or absence of FGF+AA. (**A**) Representative micrographs of differentiated BMSCs (a–h), ASCs (i–p) and TCs (q–x treated under different induction conditions (BMP-12, MIX 1, MIX 3, MIX 5) up to Day 3 and then maintained with (+) or without (−) AA and b-FGF until Day 10. Cells were stained for DCN (red), SCX (green) and DNA (Blue). Scale bar = 200 µm. (**B**) Graphs summarizing average percentage filament coverage (±SD) of ASCs, BMSCs and TCs maintained for 7 days in complete medium (y) or maintenance medium (z) after the 3 day induction phase with different combinations of growth factors.

At the end of the induction phase on day 3, media for all cells were replaced with either Low Serum Medium or Maintenance Medium. Cells were cultured with media replacements every three days until day 10 and then SCX and DCN distribution was evaluated by immunofluorescence. The use of non-supplemented Low Serum Medium resulted in a complete loss of DCN signal during the maintenance phase (day 3–10), while the presence of b-FGF and AA allowed for the maintenance of DCN expression in all cell types until day 10 (Figure 2A). These results were confirmed by the automatic quantification of DCN filaments (Figure 2B). Interestingly, the use of Maintenance Medium allowed for increased DCN expression in MIX 1 and MIX 5 treated samples at day 10, seven days after the removal of the induction media. Thus, we decided to adopt the Maintenance Medium for subsequent experiments. 

### 2.3. Basal Levels of Tendon Specific Markers in Different Cell Types

To corroborate our findings from the immunofluorescence experiments we used Real-Time PCR to detect changes in gene expression of BMSCs, ASCs and TCs. 

**Figure 3:**
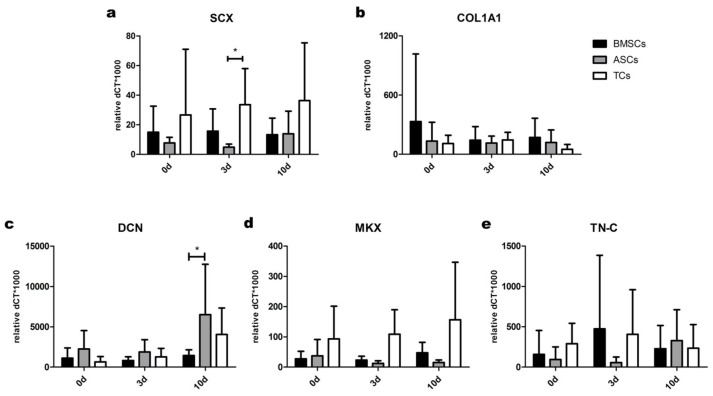
Basal expression of tendon markers in BMSCs, ASCs and TCs. Basal levels of (**a**) SCX; (**b**) COL1A1; (**c**) DCN; (**d**) MKX and (**e**) TN-C were evaluated by gene expression analysis in the three different cell types without tenogenic media supplementation. Data are expressed as mean dCT* 1000 ± SD. *n* = 7. * *p* < 0.05.

In the absence of tenogenic induction, TCs expressed the highest levels of *SCX* and *MKX* transcripts among the analyzed cell types at 0, 3 and 10 days of culture (Figure 3a,d). Moreover, the basal levels of other markers appeared to be similar in all cell populations at all analyzed time-points, with the exception of *DCN*, whose expression was higher in ASCs than BMSCs at day 10 (Figure 3c). 

### 2.4. TGF-β3 Containing Media Induce the Expression of Tenogenic Markers in TCs 

**Figure 4:**
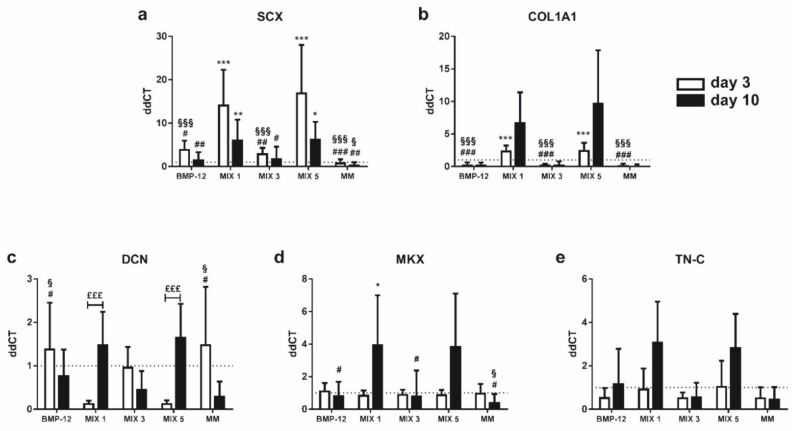
Expression of tendon-specific markers by TCs at 3 and 10 days after tenogenic induction. Expression levels of (**a**) *SCX*; (**b**) *COL1A1*; (**c**) *DCN*; (**d**) *MKX* and (**e**) *TN-C*, in TCs after tenogenic induction. Data are expressed as mean ddCT ± SD normalized to *YWHAZ* and CTRL sample. *n* = 7. * *p* < 0.05; ** *p* < 0.01; *** *p*< 0.001 vs. CTRL. ^#^ *p* < 0.05; ^##^ *p* < 0.01; ^###^ *p* < 0.001 vs. MIX 1. ^§^ *p* < 0.05; ^§§§^ *p* < 0.001 vs. MIX 5; ^£££^ *p* < 0.001, day 3 vs. day 10.

Gene expression analysis of tendon-specific markers after 3 days of tenogenic induction in TC populations revealed that levels of *SCX* and *COL1A1* were significantly increased by TGF-β3 containing media (MIX 1, MIX 5) with respect to BMP-12 and complete medium (Figure 4a,b). In contrast, TGF-β3 downregulated *DCN* expression (Figure 4c). This observation is in accordance with the results obtained from the immunofluorescence assays. 

Interestingly, the cells induced with TGF-β3 containing media showed higher *DCN*, *MKX, COL1A1* and *TN-C* expression at the end of the maintenance phase (day 10) with respect to the end of the induction phase (day 3), indicating a late effect of this growth factor on tendon marker expression (Figure 4b–e). In particular, *DCN* expression increased significantly during the maintenance phase in MIX 1 and MIX 5 (3 days vs. 10 days, *p* < 0.001). 

### 2.5. TGFβ3-Containing Media Induce the Expression of SCX in BMSCs 

**Figure 5:**
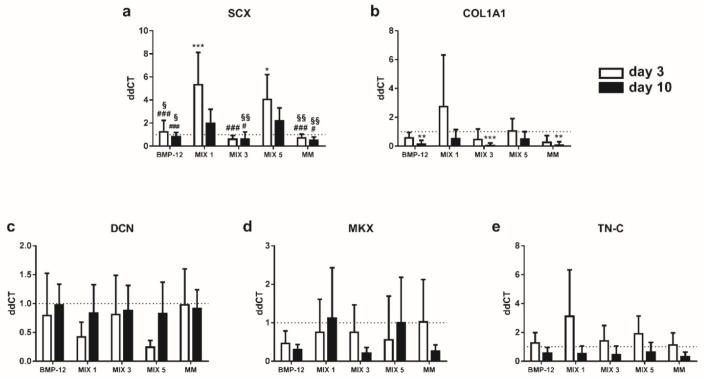
Expression of tendon-specific markers by BMSCs at 3 and 10 days after tenogenic induction. Expression levels of (**a**) *SCX*; (**b**) *COL1A1*; (**c**) *DCN*; (**d**) *MKX*; (**e**) *TN-C* in BMSCs after tenogenic induction. Data are expressed as mean ddCT ± SD normalized to *YWHAZ* and CTRL sample. *n* = 7. * *p* < 0.05; ** *p* < 0.01; *** *p* < 0.001 vs. CTRL. ^#^ *p* < 0.05; ^###^ *p* < 0.001 vs. MIX 1. ^§^ *p* < 0.05; ^§§^ *p* < 0.01 vs. MIX 5.

After three days of induction, BMSCs cultured in media containing TGF-β3 showed significantly higher expression of *SCX* with respect to complete medium and TGF-β3 free media (Figure 5a). We observed a similar effect at the end of the maintenance phase, even in the presence of a slight reduction in *SCX* expression with respect to day 3. At the same time, MIX 1 and MIX 5 treated samples showed a slight decrease in *DCN* mRNA levels at day 3 (n.s.), confirming the inhibitory role of TGF-β3 in the expression of this marker (Figure 5c). At the end of the maintenance phase, at day 10, the expression of *COL1A1* was significantly decreased in TGF-β3 free media with respect to complete medium (Figure 5b). None of the media tested induced substantial changes to the other markers at day 10.

### 2.6. TGFβ3-Free Inductive Media Reduce the Expression of COL1A1 and MKX in ASCs 

**Figure 6:**
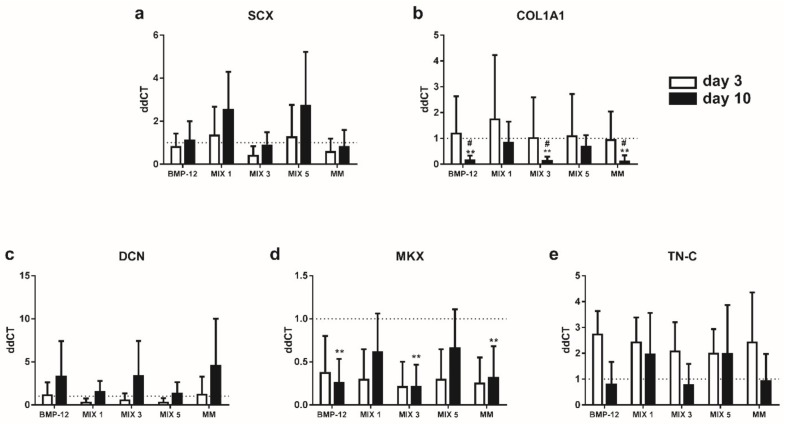
Expression of tendon-specific markers by ASCs at 3 and 10 days after tenogenic induction. Expression levels of (**a**) *SCX*; (**b**) *COL1A1*; (**c**) *DCN*; (**d**) *MKX*; (**e**) *TN-C* in ASCs after tenogenic induction. Data are expressed as mean ddCT ± SD normalized to *YWHAZ* and CTRL sample. *n* = 7. ** *p* < 0.01 vs. CTRL. ^#^ *p* < 0.05 vs. MIX 1.

None of the inductive media analyzed were able to induce a significant enhancement of tendon-specific marker expression at day 3 in ASCs (Figure 6). At day 10, a significant reduction of *COL1A1* and *MKX* expression and a slight increase of *DCN* were observed in all the samples cultured without TGF-β3 with respect to complete medium (n.s.) (Figure 6b–d). TGF-β3 containing media were able to induce a slight increase in *SCX* expression instead (n.s.) (Figure 6a).

## 3. Discussion

The present study reports a high-throughput analysis of the effects mediated by different growth factor combinations involved in the tendon differentiation of human MSCs and TCs. The results showed the early role of TGF-β3 as a key inducer of tenogenic commitment in all the cell types analyzed, as well as its later inhibitory action on collagen fiber maturation. Moreover, BMP-12, as well as CTGF and IGF-1, emerged as being subordinate to TGF-β3 in the induction of tendon-specific transcription factors, while they may be important for the production of tendon-specific extracellular matrix. TCs were the most responsive cell population in this context: although they express similar basal levels of *COL1A1*, *TN-C* and *DCN* with respect to ASCs and BMSCs, their higher expression of specific transcription factors, such as *SCX* and *MKX*, allow for a prompt response to tenogenic induction [32]. 

As mentioned above, of the growth factors tested in this study, TGF-β3 showed the most interesting properties: it induced the expression of *SCX*, the main transcription factor involved in tendon development, in all the cell types analyzed [32,35,36], while inhibiting the expression of *DCN*, a small leucine-rich proteoglycan with a crucial role in the maturation of collagen fibers [37,38,39,40]. The role of TGF-β3 in the induction of tendon progenitor markers was described in mice, rats and equine in vitro and in vivo models [41,42,43]. Likewise, the presence of a mutually inhibitory interaction between TGF-β family proteins and DCN resulting in possible interference in the process of collagen fiber maturation, has been demonstrated [44,45,46]. Taken together, these observations suggest a two-stage differentiation protocol for the generation of tendon-cells with a correctly organized extracellular matrix, comprising of an initial TGF-β3 dependent priming step followed by a matrix deposition stage in the absence of this growth factor, similar to mechanisms already reported in other contexts [43].

All the cell populations treated with TGF-β3 demonstrated the same pattern of tendon marker expression, either in the presence (MIX 1) or absence (MIX 5) of CTGF, suggesting a subordinate role for CTGF in tenogenic induction with respect to TGF-β3. Previous reports have described a role for CTGF in the enhancement of late markers of tendon tissue [31,47]. Together, these observations support a hypothesis where CTGF is involved in the tendon matrix production phase rather than in the active induction of tendon commitment. Thus, CTGF may be useful in the second step of the tenogenic differentiation protocol, rather than in the initial induction phase, similar to what was proposed by Yin and et al. in a study on rat BMSCs [43].

It is important to note that all these experiments were carried out in the presence of ascorbic acid [47,48], which is known to induce collagen expression [49,50]. As confirmation of the importance of ascorbic acid, we observed the complete loss of tendon matrix marker expression in all cell types cultured in the absence of ascorbic acid and b-FGF. Moreover, an in vivo study recently demonstrated superior tendon healing when AA was used in combination with ASCs versus ASCs alone [20]. Further studies should investigate the individual contributions of CTGF, AA and b-FGF in the production of tendon-like ECM. 

Although BMP-12 has been described as an important inducer of tendon markers in different cell types [26,33,51,52], in our experiments it appeared insufficient to drive the expression of tendon markers in MSCs or TCs. In the latter cell population, a slight induction of *SCX* was observed after treatment with TGF-β3-free/BMP-12-containing media, but it had no effect on the expression of downstream tendon markers, such as *DCN*, *COL1A1* and *TN-C*. A previous study on human TCs and ASCs showed similar results [23], demonstrating that the addition of ascorbic acid and b-FGF was insufficient to improve BMP-12 mediated induction of tendon marker expression. 

Of the cell types analyzed, TCs were the most responsive to the tenogenic induction protocol. Indeed, proper stimulation with TGF-β3 allowed for the induction of SCX and COL1A1 in the first phase of the differentiation protocol (0–3 days), while after its removal (3–10 days) the cells primed with TGF-β3-containing media showed higher levels of later tendon markers such as DCN, TN-C, MKX with respect to cells treated with TGF-β3-free media. It is important to note that none of the protocols tested in this work were able to replicate these results in human adipose- and bone marrow- derived stem cells. Although SCX induction by TGF-β3 supplemented media was visible in both cell types after 3 days of induction, no increase in tendon-specific marker expression was seen after the shift to maintenance medium, in contrast to what we observed in TCs. Nevertheless, previous studies demonstrated that genetic modifications inducing a constitutive upregulation of SCX or BMP-12 genes in BMSCs or ASCs were able to enhance the expression of tendon markers [52,53]. Since these protocols involve continuous and long-term induction of SCX or BMP-12, together with the fact that some increase in SCX was observed with our protocol, it is possible that a prolonged treatment might improve the priming of MSCs towards the tenogenic lineage. The high inter-donor variability, as well as the lack of a donor-matched harvesting of the different cell populations, represent the main limitations of the present study. In addition, the low signal observed in the high-throughput fluorescence assay for SCX staining did not allow for a clear quantification of the expression of this marker during the screening phase, limiting our understanding of the contribution of each factor to tenogenic lineage commitment. Moreover, in the present study, only the two most common sources of MSCs have been investigated, while many other tissues, such as umbilical cord or placenta, could also be used. Thus, future work towards identifying cell types best suited for tenogenic induction should compare MSCs obtained from a wider range of source tissues. Another limitation is given by not examining time-points beyond 10 days, meaning that our evaluation of the effects of the proposed protocol may not be fully comprehensive.

The importance and the novelty of this study reside in terms of the large number of growth factors and human cell types analyzed together, which are relevant to the field of regenerative medicine for tendon disorders. 

The findings obtained in this study show a crucial role for TGF-β3 as an inducer of tenogenic differentiation. However, its opposing effect as an inhibitor of fiber maturation suggests the need of a two-step protocol to achieve effective tenogenic differentiation. At the same time BMP-12, as well as CTGF and IGF-1, although inferior to TGF-β3 in the induction of tendon-specific transcription factors, may be very important in the production of tendon-specific extracellular matrix. Indeed, ascorbic acid, together with b-FGF, is of crucial importance during the matrix deposition phase, but is ineffective for the priming of cells towards the tenogenic lineage.

We observed that TCs are the population most responsive to tenogenic marker induction. Despite the low bio-availability of these cells, TCs are easily harvested from surgical waste generated by anterior cruciate ligament reconstructions with hamstring autografts. The possibility of collecting such tissues and storing them for future regenerative medicine applications deserves further investigation.

Moreover, additional studies should be conducted to define the optimal timing and dosage of tenogenic factors for tenogenic commitment in BMSCs and ASCs, as well as to clarify the roles of IGF-1 and BMP-12 in tendon matrix production.

## 4. Materials and Methods 

### 4.1. MSCs Isolation and Culture

Human BMSCs were isolated from the proximal femur of seven donors (*n* = 7, 1 male and 6 females, 57.8 ± 6.5 years old) who underwent hip replacement. Bone marrow aspirates were washed in phosphate-buffered saline (PBS) and centrifuged at 623× *g* for 10 min. The pellet was resuspended in complete medium (CM) composed of Dulbecco’s Modified Eagle Medium High Glucose (DMEM-HG, Sigma-Aldrich, St. Louis, MO, USA), 10% fetal bovine serum (FBS, Sigma-Aldrich), 50 U/mL penicillin, 50 mg/mL streptomycin, 2 mM l-glutamine (Life Technologies, Carlsbad, CA, USA) and plated in culture flasks at a density of 5 × 10^3^ cells/cm^2^. 

Human ASCs were isolated as previously described [54] from adipose tissue of eight donors (*n* = 8, 1 males and 7 females, 46.4 ± 11.3 years old) who underwent abdominoplasty. Adipose tissue was minced and washed with PBS. Samples were then digested with 0.075% *w*/*v* collagenase type I (185 U/mg, Worthington Biochemical Corporation, Lakewood, NJ, USA) at 37 °C for 30 min. The obtained stromal vascular fraction (SVF) was centrifuged (1200× *g*, 10 min) and filtered through a 100 µm nylon cell strainer (Becton, Dickinson and Company, Franklin Lakes, NJ, USA); 1 × 10^4^ cells/cm^2^ were plated in CM. 

Human TCs were isolated from fragments of *semitendinosus* and *gracilis* tendons collected from eight donors (*n* = 8, 6 males and 2 females, 33.1 ± 9.0 years old) who underwent anterior cruciate ligament (ACL) reconstruction using hamstring tendons. After 16 h of enzymatic digestion with collagenase type I at 0.3% *w*/*v* (185 U/mg, Worthington Biochemical Corporation, Lakewood, NJ, USA) [55,56], the samples were filtered through a 100 μm cell strainer (Becton, Dickinson and Co., NJ, USA) and centrifuged (300× *g*, 5 min). TCs were plated at a density of 5 × 10^3^ cells/cm^2^ in CM and maintained in an incubator at 37 °C in a humidified atmosphere with 5% CO_2_. 

All the populations were cultured until passage 4, when they were detached and seeded for the following experiments. All donors gave their informed consent for inclusion before waste surgical material collection. The study was conducted in accordance with the Declaration of Helsinki, and the protocol was approved by the Institutional Review Board (M-SPER-015- Ver. 2-04.11.2016).

### 4.2. Growth Factors-Mediated Tenogenic Induction 

At passage 4, BMSCs, ASCs and TCs were seeded at a 3000 cells/cm^2^ density and treated with different combinations of growth factors (Table 1) for three days (induction phase). All media combinations comprise HG-DMEM (Sigma-Aldrich) + 1% PSG (Penicillin-Streptomycin-Glutamine, Gibco, Waltham, MA, USA). Negative control medium (CTRL) is represented by complete medium, also containing 10% HyClone Fetal Bovine Serum (FBS, GE Healthcare, Little Chalfont, UK), while all other combinations contain low serum concentration (1% FBS). The addition of Ascorbic Acid (AA) 25 µg/mL and b-FGF 5 ng/mL completed the Maintenance Medium composition. The different tenogenic inductive media were composed of different growth factor combinations in Maintenance Medium. The following growth factors were considered for tenogenic induction: Bone Morphogenetic Protein 12 (BMP-12), Connective Tissue Growth Factor (CTGF), Transforming Growth Factor β3 (TGF-β3) and Insulin-like Growth Factor 1 (IGF-1) (Peprotech, London, UK). Table 1 lists all the different combinations. Maintenance Medium supplemented by BMP-12 alone (BMP-12) was considered as a positive control. 

After three days of tenogenic induction, media were changed to fresh Maintenance Medium or Low Serum Medium. For the negative controls, cells were cultured in complete medium (CTRL) throughout the experiment. 

### 4.3. Immunofluorescence Analysis 

To efficiently quantify tenogenic markers and morphological modifications, an immunofluorescence-based high-throughput system, Operetta^®^ (Perkin Elmer, Waltham, MA, USA) was used. Cells were seeded in 96 well plates with a polystyrene base suitable for microscopy (Thermo Fisher Scientific, Waltham, MA, USA; Ref. 165305). Cells were fixed with ice-cold 100% methanol for 5 min at room temperature and then washed with ice cold PBS. Samples were then treated with a blocking solution containing 1% bovine serum albumin (BSA, Sigma-Aldrich). Primary antibodies diluted in blocking solution (for scleraxis, SCX, rabbit anti-human, 0.5 µg/mL and decorin, DCN, mouse anti-human, 0.5 µg/mL; Abcam, Cambridge, UK) were added to the samples and incubated overnight at 4 °C. Cells were then rinsed twice with PBS + 0.1% Tween 20 and incubated with the secondary antibodies diluted in PBS (Goat Anti-Rabbit IgG H&L, Alexa Fluor^®^ 488, 2 µg/mL; Rat monoclonal (SB74g) Anti-Mouse IgG2b gamma chain, Alexa Fluor^®^ 647, 2 µg/mL; Abcam) for 1 h at room temperature. Finally, the cells were washed and incubated with 0.1 μg/mL DAPI (DNA stain, Abcam) for 1 min. Negative controls were generated by removing primary antibodies from the blocking buffer during the overnight incubation at 4 °C. Images corresponding SCX, DCN and DAPI fluorescence plus transmitted light were automatically captured using the Operetta system using a 20x LWD 0.45 NA air objective lens at multiple randomly selected fields in wells corresponding to the different treatments and replicas for each time-point. Image analysis was performed using the integrated Harmony software. Measurement of image intensity and measurement of specific tenogenic morphology were performed. Briefly, image intensity analysis was performed automatically, measuring nuclear and cytoplasmic staining intensities using DAPI staining to delimit specific regions inside and outside of the nuclei. The characteristic DCN filaments formed in differentiating tendons were identified using the Phenologic machine-learning algorithms built into the Harmony software. Briefly, small regions of DCN filaments from positive control images were manually identified to train the software to identify this particular combination of staining and subcellular organization. Following several rounds of automatic prediction, correction and validation, the specificity of the trained algorithm for detecting filament containing regions was confirmed. The amount of Decorin-filament formation is expressed as a ratio of the total image area. 

### 4.4. RNA Extraction and Gene Expression Analysis

Quantitative real time PCR was performed at days 0, 3 and 10. Total RNA was extracted using a PureLink^®^ RNA Mini Kit (Life Technologies) and reverse transcribed to cDNA (5 min at 25 °C, 30 min at 42 °C and 5 min at 85 °C) using an iScript™ cDNA Synthesis Kit (Bio-Rad Laboratories, Hercules, CA, USA). 10 ng of cDNA was used as template and incubated with a PCR mix containing TaqMan^®^ Universal PCR Master Mix and Assays-on-Demand Gene expression probes (Life Technologies) for the following genes: *SCX* (Hs03054634_g1), *DCN* (Hs00370385_m1), *COL1A1* (Collagen Type I alpha 1 chain, Hs01076777_m1), *MKX* (Mohawk Homeobox, Hs00543190_m1) and *TN*-*C* (Tenascin C, Hs01115665_m1). Reactions were performed with Applied Biosystems^®^ StepOnePlus™ (Life Technologies; 50 °C for 2 min, 95 °C for 10 min, 40 cycles at 95 °C for 15 s and 60 °C for 1 min). The fold change in expression was normalized against the expression of the previously validated housekeeping gene *YWHAZ* (Tyrosine 3-Monooxygenase/Tryptophan 5-Monooxygenase Activation Protein Zeta, Hs03044281_g1) [57]. Two replicates were analyzed for each experimental group. Data were expressed according to the dCt or ddCt method [58].

### 4.5. Statistical Analysis

Statistical analysis was performed using GraphPad Prism v5.0 software (GraphPad Software Inc., La Jolla, CA, USA). Gene expression data are expressed as mean dCT or ddCT ± SD, as indicated in each figure. Normal distribution of values was assayed by Kolmogorov–Smirnov normality test, while one-way analysis of variance (ANOVA) for repeated measures, with Bonferroni’s post-test, was used to compare the data from each group. *p* values < 0.05 were considered statistically significant.

### 4.6. Data Availability

The data used to support the findings of this study are available from the corresponding author upon reasonable request.

## Figures and Tables

**Table 1:** Tenogenic media composition with different combinations of growth factors and supplements.

Samples	Growth Factors and Supplements						
	FBS	BMP-12 (50 ng/mL)	IGF-1 (50 ng/mL)	CTGF (100 ng/mL)	TGF-β3 (20 ng/mL)	AA (25 µg/mL)	b-FGF (5 ng/mL)
BMP-12	1%	+	-	-	-	+	+
MIX 1	1%	+	+	+	+	+	+
MIX 2	1%	-	+	+	+	+	+
MIX 3	1%	+	+	+	-	+	+
MIX 4	1%	+	-	+	+	+	+
MIX 5	1%	+	+	-	+	+	+
Maintenance Medium	1%	-	-	-	-	+	+
Low Serum Medium	1%	-	-	-	-	-	-
Complete Medium	10%	-	-	-	-	-	-

Supplements present (+) or absent (-) in each medium; FBS, fetal bovine serum; BMP-12, bone morphogenetic protein 12; IGF-1, Insulin-like growth factor 1; CTGF, connective tissue growth factor; TGFβ3, transforming growth factor β3; AA, ascorbic acid; b-FGF, basic fibroblast growth factor.

## Supplementary Materials

The supplementary materials are available online at http://www.mdpi.com/1422-0067/20/1/149/s1.
