# Supplementary figures and images for: In Vitro Induction of Tendon-Specific Markers in Tendon Cells, Adipose- and Bone Marrow-Derived Stem Cells is Dependent on TGFβ3, BMP-12 and Ascorbic Acid Stimulation
Source: Int J Mol Sci. 2019 Jan 3;20(1):149. doi: 10.3390/ijms20010149 (PMC6337430; doi:10.3390/ijms20010149)

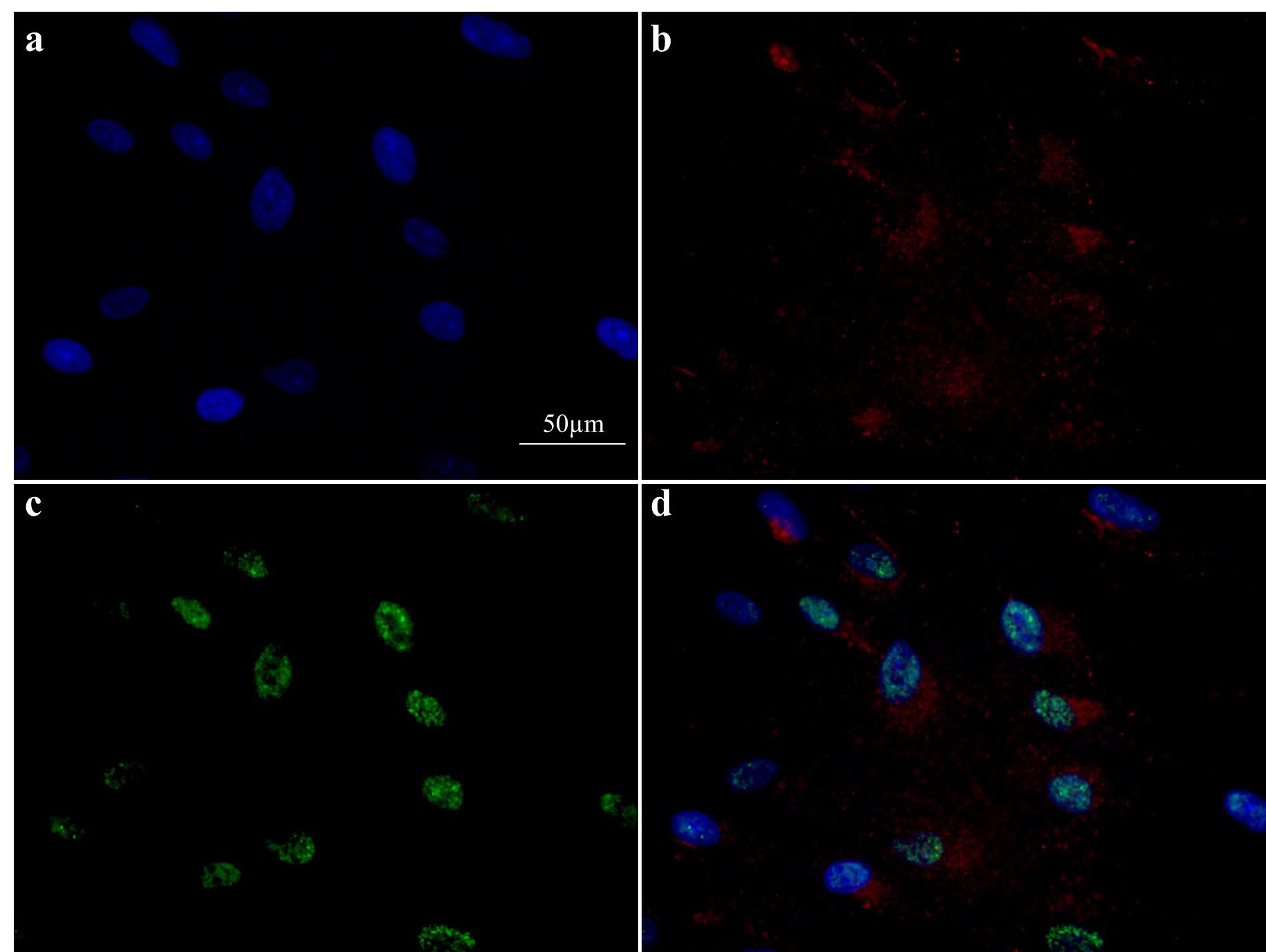

Supplement: Supplementary file 1 [file ijms-20-00149-s001.zip › Supplementary Figure 2.tiff]

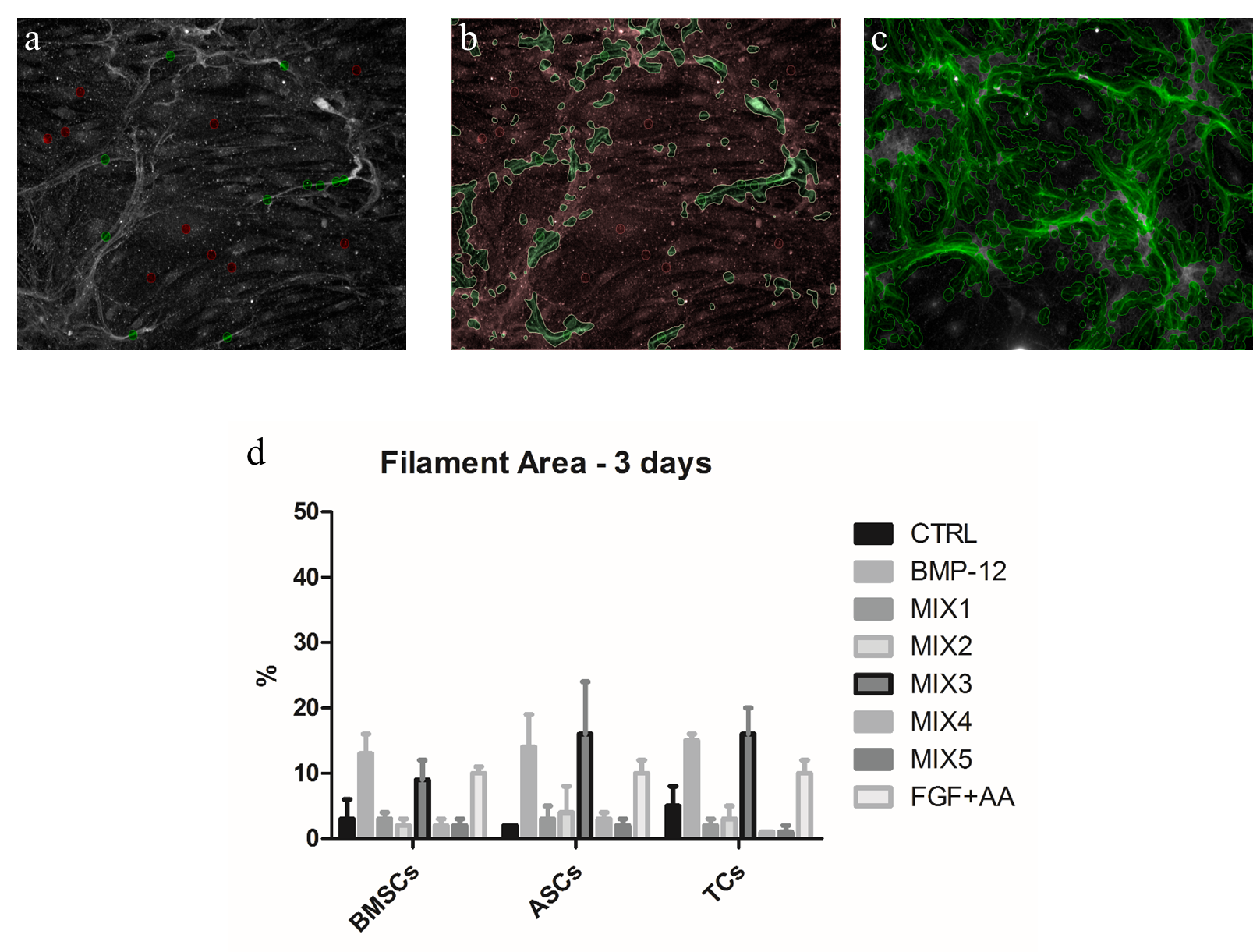

Supplement: Supplementary file 1 [file ijms-20-00149-s001.zip › Supplementary figure 1.tif]

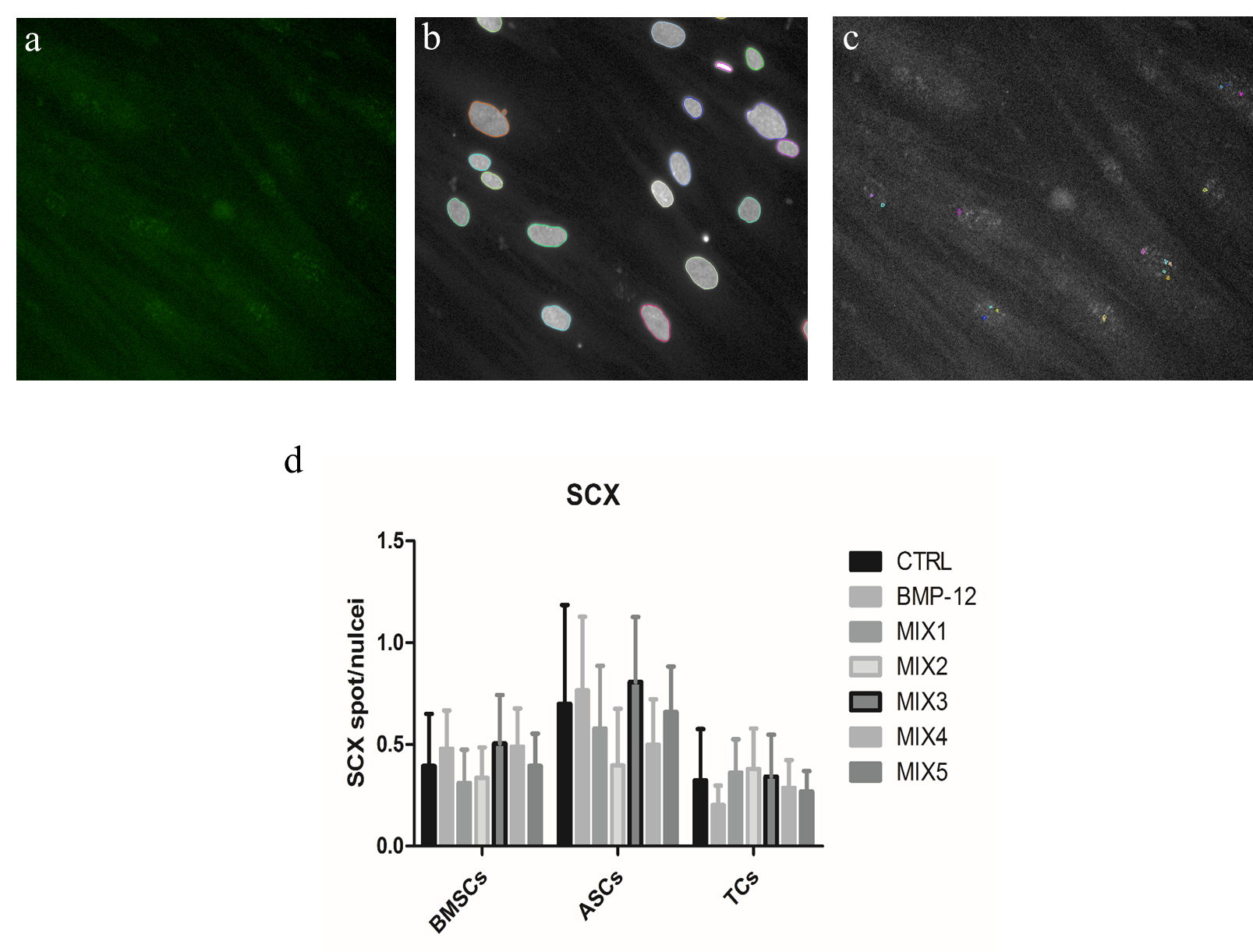

Supplement: Supplementary file 1 [file ijms-20-00149-s001.zip › Supplementary Figure 3.tiff]
